# Molecular Physiology of Anaerobic Phototrophic Purple and Green Sulfur Bacteria

**DOI:** 10.3390/ijms22126398

**Published:** 2021-06-15

**Authors:** Ivan Kushkevych, Jiří Procházka, Márió Gajdács, Simon K.-M. R. Rittmann, Monika Vítězová

**Affiliations:** 1 Department of Experimental Biology, Faculty of Science, Masaryk University, 62500 Brno, Czech Republic; 436906@mail.muni.cz; 2 Department of Pharmacodynamics and Biopharmacy, Faculty of Pharmacy, University of Szeged, Eötvös utca 6, 6720 Szeged, Hungary; gajdacs.mario@szte.hu; 3 Faculty of Medicine, Institute of Medical Microbiology, Semmelweis University, Nagyvárad tér 4, 1089 Budapest, Hungary; 4 Archaea Physiology & Biotechnology Group, Department of Functional and Evolutionary Ecology, Universität Wien, 1090 Wien, Austria; simon.rittmann@univie.ac.at

**Keywords:** molecular mechanisms of photosynthesis, anoxygenic bacteria, hydrogen sulfide, detoxification, anaerobes, water environment

## Abstract

There are two main types of bacterial photosynthesis: oxygenic (cyanobacteria) and anoxygenic (sulfur and non-sulfur phototrophs). Molecular mechanisms of photosynthesis in the phototrophic microorganisms can differ and depend on their location and pigments in the cells. This paper describes bacteria capable of molecular oxidizing hydrogen sulfide, specifically the families *Chromatiaceae* and *Chlorobiaceae*, also known as purple and green sulfur bacteria in the process of anoxygenic photosynthesis. Further, it analyzes certain important physiological processes, especially those which are characteristic for these bacterial families. Primarily, the molecular metabolism of sulfur, which oxidizes hydrogen sulfide to elementary molecular sulfur, as well as photosynthetic processes taking place inside of cells are presented. Particular attention is paid to the description of the molecular structure of the photosynthetic apparatus in these two families of phototrophs. Moreover, some of their molecular biotechnological perspectives are discussed.

## 1. Introduction

Phototrophic purple and green sulfur bacteria have been known for a long time [1]. These microorganisms are characterized by using reduced sulfur (S) compounds as electron donors in the process of anoxygenic photosynthesis and are classified into different families based on their morphology, physiological and biochemical characteristics. Representatives of the largest family, the *Chromatiaceae*—members of which may be observed in nature as a light red coloration of the anaerobic layer of water—were first described in the second half of the 19th century. In contrast, the less numerous *Chlorobiaceae* family—also referred to as green sulfur bacteria—were isolated later in the second half of the 20th century [2].

Despite the elapsed time since their first description, not all mechanisms of metabolism, anoxygenic photosynthesis and structures of these bacteria have yet been fully described. The main problem is that—although many articles have been written on this topic—there are very few scientific studies available dealing with this issue, and only a fraction of them provide comparative data on these two respective families. In most microbiological publications and monographies, the above families are mentioned only marginally or often not at all; this may be one of the reasons why the interest regarding these phototrophs from the scientific community is so small. This is best demonstrated by the fact that a large number of scientific publications dealing with these microorganisms are mainly penned by a narrow circle of scientists, including Norbert Pfennig [3], Johannes Imhoff [4] and Jörg Overmann [5].

The genomic characteristics of these bacteria (including its physiological and structural aspects) are largely unknown because they are difficult to cultivate in the laboratory. Some species of anoxygenic phototrophs can grow (cell densities of 2–5 g/L) within one week to more than one month. It was not until 2011 that the firs—and so far only—complete genome of *Allochromatium vinosum* DSM 180T, the representative of the taxon *Chromatiaceae,* was sequenced [6]. In contrast, more details are available on the *Chlorobiaceae*, as they have been better studied; by the end of 2016, 13 genomes of members of this family were fully sequenced. Nevertheless, even these developments do not cover the diversity of this family, thus necessitating further studies in this field [5].

The aim of this review is to provide a general overview of the literature available on the *Chromatiaceae* and *Chlorobiaceae* about their molecular and physiological characteristics, their phylogeny and the associated taxonomy and selected biochemical properties. Some of these properties are utilizing reduced sulfur compounds as electron donors for anoxygenic photosynthesis and the structure of their photosynthetic units, composed of light-harvesting complexes and reaction centers.

## 2. General Characteristics of the *Chromatiaceae* and *Chlorobiaceae* Families

### 2.1. Chromatiaceae

The *Chromatiaceae* family—formerly called *Thiorhodaceae* [7]—is a family defined as a group of Gamma-proteobacteria, which are—under appropriate conditions—capable of storing elemental sulfur granules within their cells [8]. Generally, these bacteria use reduced sulfur compounds (hydrogen sulfide (H_2_S)) as an electron-donor for anoxygenic photosynthesis under anaerobic conditions [9]. The vast majority of representatives are therefore anaerobic photolithoautotrophs, but there are exceptions, capable of photolithoheterotrophy, chemolithoautotrophy or chemoorganoheterotrophy at low molecular oxygen (O_2_) concentrations [10].

The specific conditions (anaerobiosis, presence of hydrogen sulfide and organic compounds) provide the growth of the species of this family [11,12]. These are mainly freshwater resources, such as lakes, ponds or pools, where these bacteria occur to a greater extent, mainly in summer and autumn, when sulfate-reducing bacteria decompose biomass and thus, increase the content of H_2_S in water [13,14,15,16]. The first mentioning of the presence of purple sulfur bacteria in these habitats dates back to the end of the 19th century [17,18]. Other frequent habitats include:

- sulfur springs (e.g., Yavoriv lake, Ukraine), mainly due to the relatively constant supply of necessary sulfur compounds [19,20],
- sources of wastewater [21] or
- the sea (e.g., Black Sea) and their shores [22,23].

Extreme habitats include hot sulfur springs (>40 °C), where *Thermochromatium tepidum* [24], the only thermophilic genus of this family may be found. Some scientists speculate that members of the *Chromatiaceae* family could exist even in sea ice [25], a speculation supported by the discovery of bacteria related to the genera *Rhabdochromatium* and *Thiorhodovibrio* [8,10,11].

**Figure 1:**
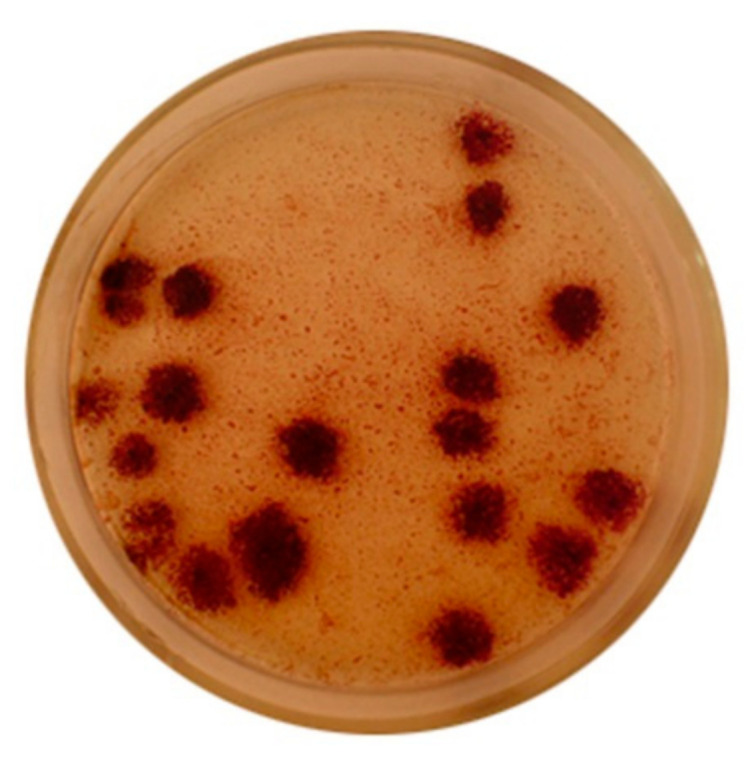
The colonies of purple sulfur bacteria of *Lamprocystis* genus [11].

Representatives of the *Chromatiaceae* family are mesophilic, predominantly motile, rod-shaped or coccoid-shaped bacteria. All representatives have bacteriochlorophyll (*BChl*) *a* or *b*, contain specific carotenoids (e.g., okenone, spiriloxanthine, lycopene). The species show low to no tolerance to O_2_ and require the presence of light and reduced sulfur compounds in the environment for growth, or other specific conditions where there is high concentration of H_2_S. The cultivation of the *Chromatiaceae* family species is difficult and it is complicated to obtain isolated colonies. Some of them appear on agar medium after 2–4 weeks of cultivation. The colonies of purple sulfur bacteria on Van Niel’s agar medium are demonstrated in Figure 1. The bacteria *Lamprocystis* sp. have been cultivated under anaerobic conditions at light intensity 500–700 lux. The purple sulfur bacteria, especially the species of *Lamprocystis* genus are interesting to study, because they can form big asymmetric conglomerates in the water environment and hence the reason for presenting the growth of their colonies below.

### 2.2. Chlorobiaceae

Members of the *Chlorobiaceae* family—sometimes referred to as green sulfur bacteria—are a phylogenetically compact and isolated group of bacteria, characterized by the ability to use reduced sulfur compounds or molecular hydrogen as an electron donor for anoxygenic photosynthesis. Another important cytological feature is the presence of elemental sulfur granules, which are stored outside of the cells by the representatives of *Chlorobiaceae* (cf. members of the *Chromatiaceae*). Microorganisms of this family are immobile, Gram-negative bacteria, whom may either be spherical, oval or rod-shaped. Some members have gas vacuoles to allow for displacement in the water column. The light intensity is more than 150 lux. All species contain special light-harvesting complexes called chlorosomes on the inside of the cytoplasmic membrane. These complexes contain bacteriochlorophylls specific for the family *Chlorobiaceae*, more specifically bacteriochlorophyll *c* or *d* in green species and bacteriochlorophyll *e* in brown species [4]. The size of these light-harvesting complexes is much bigger than that of the *Chromatiaceae* family, which allows members of the *Chlorobiaceae* family to grow at light intensities (25–80 lux) [2].

All species of the *Chlorobiaceae* family discovered so far live in aquatic environments and the majority of bacteria are mesophilic. The most common habitats include freshwater lakes, lagoons, fjords, seas and marine sediments. The only documented exception is *Chlorobium tepidum*, a thermophilic species isolated from sulfur springs, capable of growing at temperatures between 45–55 °C [26]. Because bacteria of the *Chlorobiaceae* family require anaerobic conditions for growth, a source of reduced sulfur compounds and light, they can only grow in a small area of overlap between opposing gradients of sulfur and light compounds, usually near the bottom or in the upper few millimeters of sediment. In these areas, the growth of a layer of bacteria of the family *Chlorobiaceae* growing under one or more layers of bacteria of the family *Chromatiaceae* is observed. This coexistence is especially relevant because *Chlorobiaceae* require less light intensity than *Chromatiaceae*, have a higher affinity for H_2_S as the most common source of reduced sulfur, and have less energy to maintain cell function [27]. In addition, layers of bacteria of the *Chromatiaceae* family protect *Chlorobiaceae* from O_2_, to which they have almost no tolerance. In return, they can provide extracellular elemental sulfur that reacts abiotically with sulfides to form polysulfides, which may be used immediately by members of the *Chromatiaceae* family. This syntropy has been observed, for example, among representatives of *Chlorobium limicola* and *Chromatium vinosum* [28]. Different representatives of *Chlorobiaceae* can form colonies of different shapes, often also forming so-called phototrophic consortia, communities of green sulfur bacteria with chemotrophic bacteria. These consortia are considered to be the most perfect symbiotic prokaryotic groupings ever discovered [2].

## 3. Phylogenetic and Taxonomy

### 3.1. Chromatiaceae

The first taxonomic system, including the family known today as *Chromtiaceae*, was created on the basis of the Molisch pigment and sulfur granules in 1907. The taxonomic designation *Chromatiaceae* was first used by Bavendamm in 1924, and included all bacteria using sulfides as an electron donor in photosynthesis and accumulating sulfur globules either inside or outside the cell, which corresponds not only to today’s conception of this family, but also includes the family *Ectothiorhodospiraceae* [29]. This was first set aside by Imhoff in 1984, on the basis of ecological conditions, 16S rRNA analysis, lipid composition, different quinone structures and the amino acid sequence of cytochrome *c*_551_, creating two separate and well-defined families *Chromatiaceae* and *Ectothiorhodospiraceae*. The name of the latter family was derived from the genus *Ectothiorhodospira* [8]. However, even after returning to the original definition of the family and the separation of *Ectothiorhodospiraceae*, the family *Chromatiaceae* also did undergo systematic changes. This was mainly due to the fact that the initial systems were based on morphological and phenotypic features [7,29], which—as was later found—have little or no relevance in phylogenetic relationships [30]. With the later development of chemotaxonomic and sequencing methods that have provided new evidence regarding phylogenetic relationships, the systematic classification of the *Chromatiaceae* family, its genera and species has undergone extensive changes. Important indicators of phylogenetic relatedness include lipid and fatty acid composition, with polar lipid composition being most accurate [31,32,33]; other important methods for their discrimination include the content of cytosine and guanine bases in DNA expressed as a molar fraction [32].

This ratio is only used to distinguish the organisms at the order level but not on the species level, due to variation between species and sometimes strains. Currently, the most widespread method of phylogenetic analysis is the nucleotide sequence of 16S rDNA ribosomal subunits. This method analyzes a component of a small ribosomal subunit that contains not only regions highly conserved for all microorganisms, but also regions that are variable and characteristic of individual species and strains [30,34,35,36]. The last important method of phylogenetic analysis is gene sequencing. These are mainly the *pufLM* genes, which are located on the *puf* operon. This *puf* operon encodes the genes for the structural proteins of the type II photosynthetic reaction center. In all five known types of *puf* operon, we know the order and arrangement of genes that do not change between different types of *puf* operons. The *pufL* and *pufM* genes, which encode the light and medium polypeptide chains of the photosynthetic reaction center, are highly conserved and for this reason are considered important phylogenetic markers [37].

According to the latest edition of the Bergey’s Manual of the Systematics of Archaea and Bacteria, the family *Chromatiaceae* is listed as follows:

Domain: *Bacteria*

   Phylum: *Proteobacteria*

      Class: *Gammaproteobacteria*

        Order: *Chromatiales*

          Family: *Chromatiaceae*

The *Chromatiaceae* contains more than twenty orders divided into three branches. The first group consists of halophilic and marine genera *Marichromatium*, *Thiorhodococcus*, *Thiophaeococcus*, *Halochromatium*, *Thiohalocapsa*, *Thiorhodovibrio*, *Rhabdochromatium*, *Isochromatium*, *Thiococcus*, *Thioflavicoccus* and *Thioalcalicoccus*. In some literature, this branch was divided into two groups, where the first branch consists of marine families moving by means of a whip, i.e., *Marichromatium*, *Thiorhodococcus* and *Thiophaeococcus* [10]; while the second branch was then formed by the remaining genera. Another group consists of freshwater genera moving by means of a whip, which do not have gas vacuoles. This group includes the genera *Allochromatium*, *Thermochromatium*, *Thiocystis* and *Chromatium*. The last group consists of the genera *Thiocapsa*, *Thiolamprovum*, *Thiobaca*, *Lamprocystis* and *Thiodictyon*, primarily freshwater genera, which, however, show a certain degree of tolerance to salt [10].

**Figure 2:**
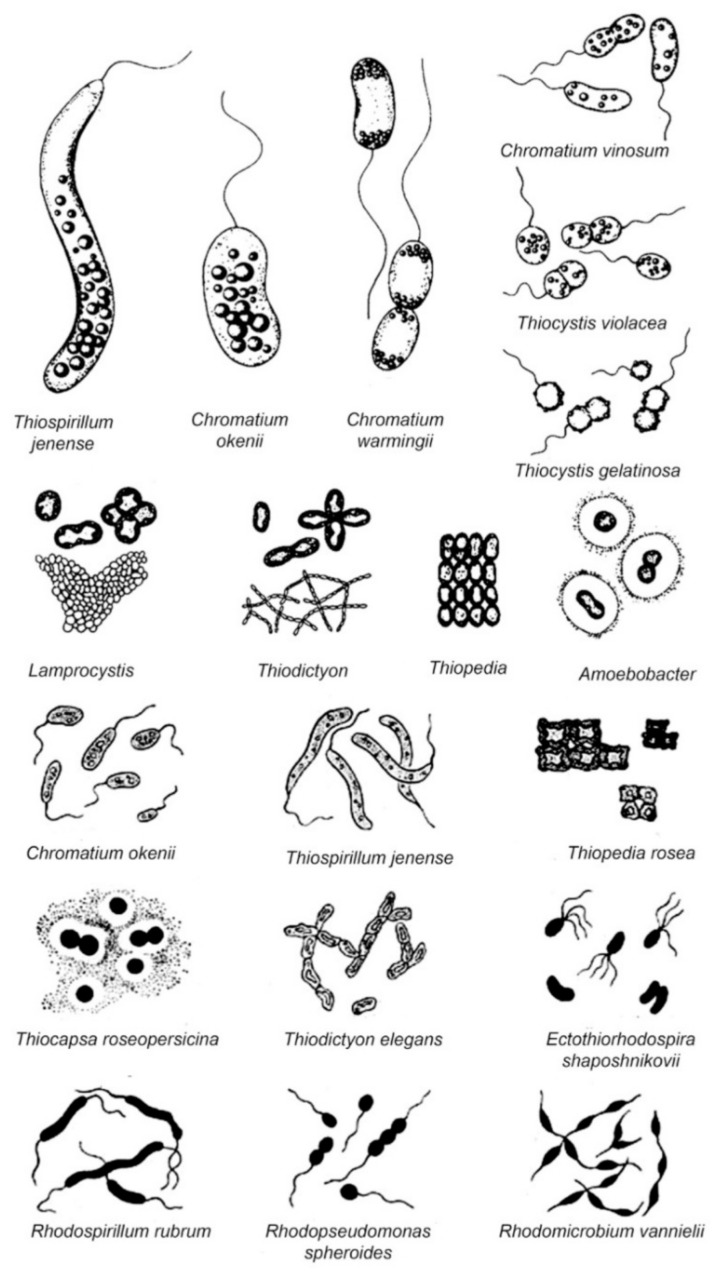
The morphological diversity of some phototrophic purple bacteria [11].

Another frequently mentioned division is according to the ability to obtain energy by means other than using phototrophy. This division has two groups: the first group are specialized species, dependent on a strictly anaerobic environment and are obligate phototrophs capable of photoassimilate only pyruvate or propionate in the presence of sulfide and CO_2_. Representatives of this group include *Chromatium okenii*, *Chromatium wissei*, *Allochromatium warmingii*, *Isochromatium buderi*, *Thiospirillum jenense* and *Thiococcus pfenigii*. The second group are capable species and photoassimilate many diverse organic substrates. Most of these species are able to grow in the absence of reduced sulfur compounds and use organic substrates as electron donors for photosynthesis. Some species of this group are even able to grow chemoautotrophically or chemoheterotrophically. This group includes *Allochromatium vinosum*, *Thiocystis violacea*, *Thiocapsa roseopersicina*, *Thiocapsa rosea*, *Thiocapsa pendens* and *Lamprobacter modestohalophilus* [10,38]. The morphological diversity of photothrophic bacterial cells, the most common in the aquatic environment has been described previously and presented in Figure 2.

If we take a closer look at the representatives of both groups, it is clear that this division has no significance in terms of phylogeny, and thus the ability to use another source of electrons or obtain energy other than autophototrophically, is phylogenetically and taxonomically insignificant. In contrast, if we look at the previous division into three branches, we can conclude that the ability to grow at certain salt concentrations in the environment is phylogenetically relevant.

### 3.2. Chlorobiaceae

Since its discovery and first description by Larsen in 1953, this family has gained the attention by scientists, especially due to its unique features, such as the structure of the photosynthetic apparatus and the presence of chlorosomes, which are small organelles serving as light-collecting antennas [1,2]. Among the most important scientists is Norbert Pfennig [3], who isolated and characterized many strains and created a taxonomic system based on morphological and phenotypic features. Characteristics used for taxonomic classification included cell morphology, pigment composition, absorption spectra and certain metabolic properties. These properties were mainly:

- the composition of carotenoids and bacteriochlorophyll, for the division of species into green and brown,
- the ability to form gas vacuoles, to distinguish between genera and the ability to use thiosulfates as an electron donor for photosynthesis, to distinguish subspecies [3,39].

Although these are easy distinguishable traits allowing a clear taxonomic distribution, it has been shown that these traits are not phylogenetically relevant, especially when broken down at species and strain level [5,40].

The first phylogenetic analyzes were performed in the mid-1980s. The first work describing the phylogenetic relationships of members of the *Chlorobiaceae* family based on the oligonucleotide sequence of 16S rDNA, was published by Gibson et al. in 1985. This work dealt with phylogenetic relationships between members of the family *Chlorobiaceae* and members of the genus *Chloroflexus*. The study confirmed that the family *Chlorobiaceae* forms a relatively phylogenetically isolated group and also provided evidence of phylogenetic separation from the genus *Chloroflexus*, which also carries chlorosomes [41].

Further pivotal work from the same year, analyzing approximately 400 bacterial 16S rDNAs, aimed to identify the major branches of the phylogenetic tree and tried to determine specific positions in the oligonucleotide sequence for each of these branches. The result of this study was the description of 10 separate branches, one of which was a branch of green sulfur bacteria, i.e., family *Chlorobiaceae*. More important, specific sequences characteristic to this group were also identified [42].

In 1997, Overmann and Tuschak investigated the possibility of distinguishing individual species of the *Chlorobiaceae* family, based on their 16S rDNA sequence. This work identified base changes mainly in the variable regions V2 to V8, with the V5 region showing only very small differences across the species studied. Based on these results, the first phylogenetic tree was compiled, which showed some inconsistencies in classical taxonomy [5].

In 2002, in addition to sequencing the 16S rRNA molecule, the gene for the Fenna–Matthews–Olson protein (FMO) was also sequenced. It is a water-soluble protein that binds 8 molecules of bacteriochlorophyll and is responsible for energy transfer between chlorosomes and the reaction center [43]. The FMO protein occurs in the form of a trimer, but only the *fmoA* gene, which encodes one of the monomers, has been sequenced. This protein was used mainly because it is specific for green sulfur bacteria and is not found in the genus *Chloroflexus* [5]. Based on these results, two phylogenetic trees were constructed, one according to the 16S rDNA sequence and the other according to the amino acid sequence of the FMO protein [43]. The topology of phylogenetic trees has been shown to be basically the same, which confirms the accuracy of phylogenetic analyzes of 16S rDNA compared to the current system based on physiological and morphological features. Based on these analysis, species of the family *Chlorobiaceae* may be divided into 5 groups [43].

The first group consists of strains *Prosthecochloris aestuarii* DSM 271T and 2K, *Chlorobium vibrioforme* DSM 260T and *Chlorobium phaeovibrioides* DSM 1678. This is the most diverse group consisting of purely marine species. The second group is made up of vibrio-shaped bacteria, which need small amount of salt (1% NaCl) to grow. This group includes strains *Chlorobium vibrioforme* DSM 261 and DSM 262, *Chlorobium vibrioforme f. thiosulfatophilum* DSM 265T, *Chlorobium phaeovibrioides* DSM 269T and DSM 270 and *Pelodictyon luteolum* DSM 273T. The third group is a rod-shaped species occurring in freshwater. These include *Chlorobium ferrooxidans* DSM 13031T, *Pelodictyon clathratiforme* PG, *Chlorobium phaeobacteroides* DSM 266T, DSM 1855 and UdG 6051, *Chlorobium limicola* DSM 245T and DSM 246, *Pelodictyon phaeoclathratiforme* DSM 5477T and *Chlorobium limicola f. thiosulfatophilum* 1630 and 9330. The fourth group consists mainly of freshwater strains of *Chlorobium tepidum* ATCC 49652T, *Chlorobium limicola* UdG 6041, *Chlorobium limicola f. thiosulfatophilum* 1430 and DSM 249T and *Chlorobium phaeobacteroides* 1549 and DSM 1677, but there are also strains with low salt requirements, such as *Chlorobium chlorovibrioides* UdG 6026 and *Chlorobium vibrioforme f. thiosulfatophilum* DSM 263 and NCIB 8346 [43,44,45]. The fifth group consists of a single species of *Chloroherpeton thalassium* and is the most isolated group in the family.

The phylogenetic analysis and the phenotypic traits are important for taxonomy of these bacteria. These characteristics are, as in the case of the *Chromatiaceae* family, the ratio of cytosine and guanine bases in DNA, fatty acid composition and salt content requirements in the environment. Another feature is the ability to triple division (from the English “ternary fission”), i.e., the ability to divide into three cells during division instead of the classic two [2].

As mentioned at the beginning of the chapter, this family taxonomic system was primarily based on easily recognizable phenotypic traits that do not represent phylogenetic relationships, although several 16S rDNA and FMO protein analysis were performed to provide a phylogenetically representative taxonomic system. The publication of Bergey’s Manual of the Systematics of *Archaea* and *Bacteria* still maintains a taxonomic system based on phenotypic traits. This system ranks the family *Chlorobiaceae* as follows:

Domain: *Bacteria*

   Phylum: *Chlorobi*

     Class: *Chlorobia*

       Order: *Chlorobiales*

          Family: *Chlorobiaceae*

The family consists of 5 genera and 14 species. The genera are divided on the basis of morphology cells, motility and ability to form gas vacuoles, while species are divided according to their own morphology and composition of the pigment [2].

## 4. Molecular Mechanisms of Sulfur Metabolism in Phototrophic Bacteria

Reduced sulfur compounds, such as sulfides (S^2−^) and thiosulfites (O_2_S_2_^2−^), are oxidized by a large and diverse group of prokaryotes, including phototrophic sulfur bacteria, thiobacilli, and other chemotrophic sulfur bacteria, or even thermophilic ones *Archaea* [46]. These compounds are usually oxidized to sulfates, but in many cases, they form polymeric water-insoluble sulfur granules as a by-product of metabolism. These granules may be found inside (in the case of purple sulfur bacteria of the *Chromatiaceae* family) or outside the cell (in the case of green sulfur bacteria of the *Chlorobiaceae* family) [11]. It should be noted at the outset that most of the following examples of sulfur oxidation in anaerobic sulfur bacteria are described for the species *Allochromatium vinosum*, whose sulfur metabolism is best studied [27], hence the reason for choosing the model to conduct the review and to illustrate the information.

### 4.1. Oxidation of Sulfide to Elemental Sulfur

There are two main metabolic pathways for the oxidation of H_2_S to S^0^, which is subsequently stored in or out of the cell. The first is via flavocytochrome c, the second is via sulfide: quinone oxidoreductase (SQR for short). Another oxidation option is the Sox enzyme system or reverse-operating sulfite reductase [46].

#### 4.1.1. Flavocytochrome *c*

Flavocytochrome *c* is a periplasmic protein consisting of two monomers. One monomer is the larger, FAD-binding, FccB subunit and the smaller is a heme-binding, FccA subunit. In vitro, flavocytochromes can effectively catalyze electron transfer between sulfides and a number of smaller cytochromes of type *c*, such as cytochrome *c*_550_, which can then further transfer electrons to the photosynthetic reaction center [47]. However, the in vivo role of flavocytochrome ***c*** is not entirely clear for several reasons. The first reason is that many organisms that use sulfides as an electron donor produce flavocytochrome ***c***, there are those that do not produce it and still use sulfides, proving that there are other ways to oxidize sulfides [48]. Another reason is that mutants of *Allochromatium vinosum* and *Chlorobium tepidum* not producing flavocytochrome ***c*** show very similar rates of sulfide oxidation as non-mutated controls [46]. The last evidence is the oxidation of sulfides, which results in the formation of elemental sulfur granules, further oxidized to sulfites in the absence of sulfides. This process occurs not only in the strain *Chlorobium limicola* DSMZ 245T, which produces flavocytochrome *c*, but also in the strain *Chlorobium luteolum* DSM 273T, which does not have flavocytochrome *c*. From these examples it should be clear that, although some members of the *Chlorobiaceae* and *Chromatiaceae* families use flavocytochrome ***c*** to oxidize sulfides, this is not the primary way to do so [49].

#### 4.1.2. Sulfide: Quinone Oxidoreductase

An alternative to oxidation with flavocytochrome *c* is the transfer of electrons from the sulfide to the quinone pool in the cell. This requires the enzyme SQR, which catalyzes the oxidation of sulfide and uses isoprenoid quinone as an electron acceptor. This mechanism has been discovered not only in chemotrophic and phototrophic prokaryotes, but even in some mitochondria [50,51]. The membrane-bound activity of the SQR enzyme has been biochemically demonstrated in both purple and green sulfur bacteria [52]. This enzyme is thought to carry electrons into the photosynthetic electron transport chain via a complex of quinol-oxidizing Reiske FeS protein and cytochrome *b* [53]. Homologues of the enzyme SQR are found in all strains of green sulfur bacteria, including *Chlorobium ferrooxidans*, the only member of the family *Chlorobiaceae* that does not use sulfur compounds as an electron donor. However, the importance of these enzymes is not fully known, mainly due to the fact that mutant *Chlorobium tepidum* with an inactivated *sqr* gene show reduced sulfide oxidation values, leading to the conclusion that these organisms have an alternative route of sulfide metabolism [46]. Some representatives even have SQR homologues that have no obvious function, they are SQRLP1 homologues and SQRLP2. Another interesting fact is that although *Allochromatium vinosum* membranes show SQR activity, its homolog of the *sqr* gene has not yet been discovered (this gene would have been originally discovered and described in *Rhodobacter capsulatus*). From the information presented above, it can be deduced that the sequence of *sqr* genes is not very well preserved and it can be assumed that these are highly variable sections of the genome, which makes their detection considerably more difficult.

The primary product of the in vitro enzymatic reaction of SQR is a water-soluble polysulfide, while elemental sulfur is not produced. Thus, it is most likely that disulfides or longer chains of polysulfides are the initial product of the enzymatic reaction, which subsequently released from the enzyme [54]. Polysulfide anions with chains of different lengths can then form longer chains by means of a disproportionate reaction. In principle, elemental sulfur is formed spontaneously [55].

#### 4.1.3. The Sox Enzyme System and Reverse Operating Sulfite Reductase

The Sox enzyme system is still best described for *Rhodobacter capsulatus*, but *sox* genes for these proteins have also been discovered in *Allochromatium vinosum*. However, it is necessary to add that mutant representatives of *Allochromatium vinosum* deficient in flavocytochrome ***c***, *sox* genes, or both, still showed no significant difference of sulfide oxidation values as controls. From this, it can be concluded that the SQR pathway is the primary pathway of oxidation of sulfide to elemental sulfur in *Allochromatium vinosum*.

Last but not least, the catabolic enzyme sulfite reductase (DsrAB) is operating a biologically-reverse function. This means that instead of decomposing sulfites into sulfides, it assimilates sulfides to form sulfites. Such a functioning protein has been found in *Allochromatium vinosum*, but it has been found that this protein is not essential for sulfide degradation but is essential for the oxidation of intracellularly deposited sulfur [56,57].

### 4.2. Oxidation of Polysulfides

Polysulfides are the primary product of sulfide oxidation in most purple and green sulfur bacteria, but some may accept them from the environment. The use of such externally supplied polysulfides was investigated mainly in *Allochromatium vinosum* and *Thiocapsa roseopersicina*. Both of these organisms can utilize externally supplied polysulfides as electron donors for NAD synthesis [58,59,60]. The mechanism of polysulfides conversion into elemental sulfur granules in these microorganisms is still unknown. Theoretically, this can occur by spontaneous chemical conversion of longer polysulfides to polysulfates [59]. However, in the studies of *Allochromatium vinosum*, large amounts were not found in elemental sulfur granules cyclic sulfur concentrations and are thought to be formed by long sulfur chains with organic residues at both ends [61,62]. It is therefore believed that these organic sulfur compounds are formed by a hitherto unknown enzymatic process.

### 4.3. Intake and Oxidation of Elemental Sulfur from the Environment

Many representatives of purple and green sulfur bacteria—including model organism *Allochromatium vinosum*—are able to absorb and oxidize externally supplied elemental sulfur, but this process is not fully understood. Elemental sulfur tends to be in the environment chain, and therefore occurs in several different allotropes: most often chains of different lengths (polymer sulfur) or its cyclic form (the most stable is α-S8) [63]. However, all these allotropes have one thing in common, are hydrophobic and sparingly soluble in water, which makes it very difficult to metabolize these compounds [64].

Enzymes catalyzing uptake and oxidation have not yet been discovered in the *Chlorobiaceae* family, but members of this family are thought to use one of two main strategies. The first possibility is due to the physical contact of the cell with the insoluble substrate and the direct transfer of electrons from the cell surface to the substrate via the outer membrane proteins [65]. The second method is the excretion of reductants, such as low molecular weight thiols, which may act on substrates distant from the cell [66]. The first method of oxidation has already been described, for example, in the bacterium *Acidithiobacillus ferrooxidans*, which in this way catalyzes the attachment of sulfur to extracellular lipopolysaccharides [67]. This method of sulfur oxidation is also used by the model organism *Allochromatium vinosum*, which has been experimentally proven to require close contact of the cell with extracellular sulfur [68]. The first step during the oxidation of extracellular elemental sulfur is its accumulation in the form of intracellular granules, which are further oxidized to sulfates. Using the XANES method (“X-ray absorption near edge structure”), it was proved that *Allochromatium vinosum* uses only polymeric sulfur in the form of chains and is unlikely to utilize its cyclic form [66,68].

In green sulfur bacteria, specifically in *Chlorobaculum limnaeum*, structures have been discovered on the surface of the cell wall (so-called “spinae”). These structures are thought to mediate cell wall adhesion to the extracellularly deposited network. The presence of large capsules observed in *Chlorobaculum limnaeum*, accompanied by a large number of “spinae”, lead scientists to believe that these structures stabilize the capsules and together play an important role in the metabolism of extracellular elemental sulfur [69]. Sulfur-related genes associated with sulfur-induced outer membrane proteins (OMPs) have been identified in 10 species of green sulfur bacteria whose genome has been fully sequenced and which are able to metabolize externally supplied elemental sulfur. These are proteins discovered in *Acidithiobacillus ferrooxidans*, responsible for the mobilization of extracellular elemental sulfur and its transfer into the periplasmic space [70].

### 4.4. Oxidation of Accumulated Sulfur to Sulfites

Oxidative degradation of sulfur in granules is currently the main focus of research on phototrophic sulfur bacteria and sulfur metabolism, but many questions still remain on this topic. In addition, in the case of extracellularly stored sulfur in the family *Chlorobiaceae*, this process must also involve binding, activation and transport into the cell.

The only known region of the genes that is essential for the oxidation of sulfur granules is the region of 15 reading frames designated as *dsrABEFHCMKLOPNRS* described in *Allochromatium vinosum* DSMZ 180T, *Halorhodospira halophila* SL1, *Chlorobium phaeovibrioides* DSMZ 265 and *Chlorobaculum tepidum* TSL. Genes marked with the same number are homologous. The first two genes (*dsrAB*) encode the reverse-operating catabolic enzyme sulfite reductase previously mentioned.

**Figure 3:**
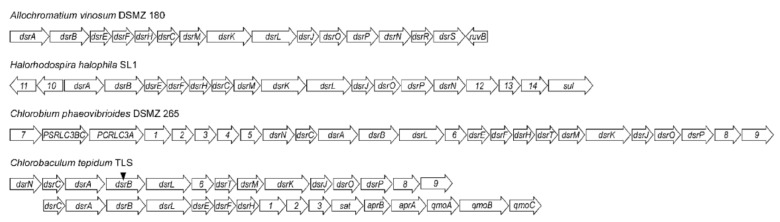
Schematic comparison of the *dsr* gene region arrangement found in *Allochromatium vinosum* with another species of phototrophs (data from Frigaard and Dahl, 2009 [46]).

In *Allochromatium vinosum*, the products of the *dsrAB* genes form the cytoplasmic α2β2 structure of sulfide reductase. The prosthetic group of *DsrAB* is siroamide- [Fe4S4], which is amidated by sirohem [57]. A very similar grouping of genes can be found in *Halorhodospira halophila* and most members of the *Chlorobiaceae* family. These have a cluster of dsrNCABLEFHTMKJOP genes, the only differences from which found in the region found in *Allochromatium vinosum* are the absence of *dsrRS* genes and the presence of the *dsrT* gene (Figure 3).

These clusters presented in Figure 3 were found in all representatives except *Chlorobium ferrooxidans* and *Chloroherpeton thalassium*. The absence of this region is particularly interesting in *Chloroherpeton thalassium*, as this bacterium oxidizes sulfides to form elemental sulfur, which it stores outside the cell. However, this sulfur is only oxidized very slowly, which is probably due to the absence of the Dsr system. The alternative route of oxidation of sulfur granules is not yet clear, but the most likely is the involvement of RuBisCO-like protein (RLP), which is found in all representatives of green sulfur bacteria [71].

**Figure 4:**
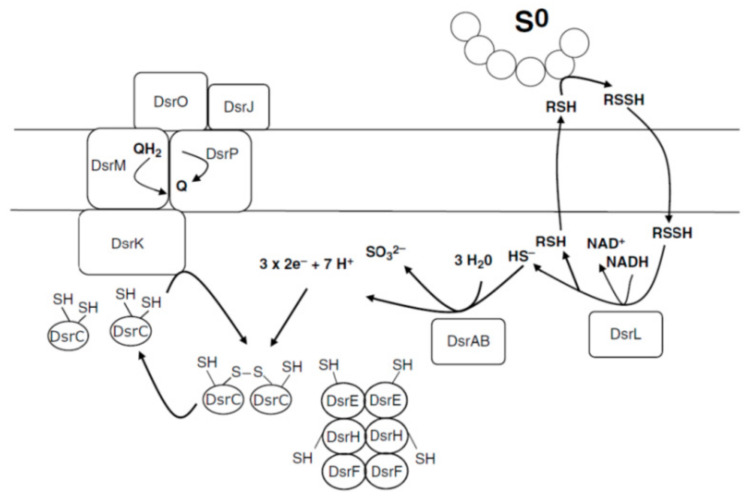
Schematic distribution of Dsr proteins in the Allochromatium vinosum cell. This scheme is based on sequence analysis of dsr coding genes and on available biochemical information (data from Dahl, 2008 [66]).

Another group of genes is *dsrEFH* located next to the *dsrAB* genes. The products of these genes are very similar and are soluble cytoplasmic holoproteins. The DsrC protein is a small soluble protein with two highly conserved cysteine residues at the C-terminus. DsrEFH and DsrC-like proteins have been shown to act in the sulfur transport system of *Escherichia coli* and are therefore thought to be preserved in other organisms [72,73]. The protein encoded by the *dsrM* gene is most likely a membrane cytochrome type *b* resembling the heterodisulfide reductase subunit found in methanogenic archaea [74]. DsrK iron-sulfur protein is most likely to occur in the cytoplasm and also bears resemblance to the heterodisulfide reductase catalytic subunit [57,75]. DsrP is an integral membrane protein and the DsrJ and DsrO proteins are a tritochrome ***c*** and an iron–sulfur protein found in the periplasm. All three of these proteins co-purified from *Allochromatium vinosum*, suggest their involvement in transmembrane electron transfer (Figure 4; [6]).

### 4.5. Oxidation of Sulfites to Sulfate

Oxidation of sulfites to sulfates is the last step in the oxidation of sulfur compounds in phototrophic sulfur bacteria. While green sulfur bacteria are generally unable to oxidize externally supplied sulfites, some purple sulfur bacteria have this ability. In addition, sulfites occur in the cytoplasm of all phototrophic sulfur bacteria as an intermediate in sulfur metabolism by the Dsr system. So far, only two methods of oxidation of sulfites are known [76]. The first method is direct oxidation by sulfite dehydrogenase, the second method is indirect AMP-dependent oxidation via adenosine-5′-phosphosulfate (APS). It has been enzymatically demonstrated that both pathways can be found in one organism [77].

#### 4.5.1. Direct Oxidation by Sulfite Dehydrogenase

Sulfite dehydrogenase, which catalyzes the direct oxidation of sulfites to sulfates, is characterized by the ability to transfer electrons to ferricyanides or flavocytochrome ***c*** [76]. The best described bacterial sulfite dehydrogenase is the SorAB protein, which is derived from the alpha-proteobacterium *Starkeya novella*. It is a periplasmic heterodimer composed of a large molybdenum cofactor and a small cytochome. During catalysis, electrons are gradually transferred by *c*_552_ hem subunit located in a small subunit, from where they are further transferred to the cytochrome *c*_550_, which is considered to be the innate electron acceptor of this enzyme [78]. However, sorAB-related genes are not found in the genomes of purple and green sulfur bacteria described so far, but are thought to have other genes performing a similar function [46].

#### 4.5.2. Indirect AMP Dependent Oxidation

During indirect oxidation of sulfites, APS is formed from sulfite and AMP by APS reductase. In the second step, the AMP portion of APS is transferred either to pyrophosphate by ATP sulfurylase or to phosphate by adenyl sulfate: phosphate adenylyltransferase (APAT), leading to create an ATP or ADP. Because two molecules of ADP can be converted to ATP and AMP using adenylate kinase, both enzymes (ATP sulfurylase and APAT) catalyze the phosphorylation of the substrate, which releases the sulfate anion [66]. The whole process takes place in the bacterial cytoplasm, with APS being either membrane-bound (for example in most *Chromatiaceae*) or dissolved in the cytoplasm, while ATP sulfurylase and APAT are exclusively soluble [79]. In *Allochromatium vinosum*, the genes for ATP sulfurylase (*sat*) and APS reductase (*aprMBA*) are in the form of an operon, while in the genomes of 4 green sulfur bacteria, these genes are directly adjacent and always grouped with Qmo complex genes (*qmoABC*), which is a membrane bound electron-transfer complex [80,81].

### 4.6. Oxidation of Thiosulfates

Thiosulfate (chemical formula: S_2_O_3_^2−^) is an oxyanion of sulfur and the prefix thio- indicates that the thiosulfate ion is a sulfate ion with one oxygen replaced by sulfur. They occur naturally and are produced by certain biochemical processes. Thiosulfates are relatively stable and abundant substances. So far, two pathways are known by which bacteria oxidize thiosulfates: the first route is oxidation to tetrathionane using thiosulfate dehydrogenase, while the second possibility is its oxidation to sulfates using the multienzyme Sox system [82].

#### 4.6.1. Oxidation of Thiosulfates to Tetrathionate

This metabolic pathway is known in only a few purple sulfur bacteria, including *Allochromatium vinosum* [82]: the ratio of conversion to tetrathionate and sulphates is pH dependent, with tetrathionate being more produced in a weakly acidic environment. The conversion is caused by the enzyme thiosulfate dehydrogenase (pH_opt_ = 4.25). It is a periplasmic monomer containing the heme ***c*** subunit. This enzyme uses HiPIP (high potential iron-sulfur protein), which is also found in the periplasm, as an acceptor of electrons released during the oxidation of thiosulfates [83].

#### 4.6.2. Oxidation of Thiosulfates to Sulfates

Many purple and green sulfur bacteria can oxidize thiosulfates to sulfates, using the *sox* genes for the periplasmic thiosulfate oxidizing the Sox multienzyme complex. All green sulfur bacteria, in which the Sox complex has been discovered, contain a group of *sox* genes soxJXYZAKBW [80]. *Allochromatium vinosum* also contains sox genes. Using gene inactivation and complementation assays, the soxBXA and soxYZ genes, located in two different regions of DNA, have been found to be essential for the oxidation of thiosulfates [84].

Thus, summarizing the section of molecular mechanisms of sulfur metabolism in phototrophic bacteria, we can conclude that sulfite is a well-established intermediate during reduced sulfur compound oxidation. Sulfite is generated in the cytoplasm by the reverse-acting dissimilatory sulfite reductase DsrAB. The inhibition of sulfite oxidation by tungstate in the model organism *Allochromatium vinosum* indicated the involvement of a molybdoenzyme, but homologues of the periplasmic molybdopterin-containing *SorAB* or *SorT* sulfite dehydrogenases are not encoded in genome-sequenced purple or green sulfur bacteria. However, genes for a membrane-bound polysulfide reductase-like iron–sulfur molybdoprotein (SoeABC) are universally present. More information about the sulfite oxidation and genes related is described in detail in the work by Dahl et al. (2013) [85].

## 5. Photosynthetic Apparatus

### 5.1. Light-Harvesting Complexes of the Chromatiaceae

**Figure 5:**
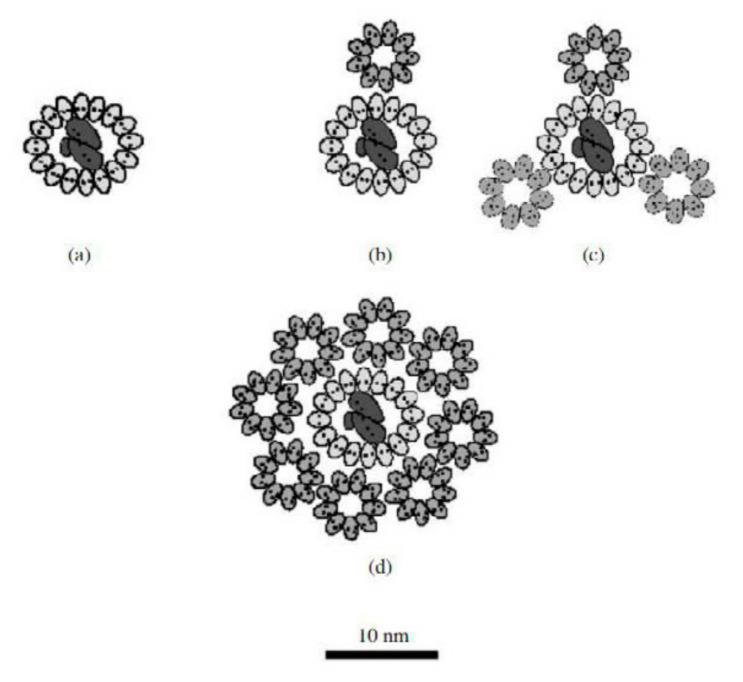
Model of a possible arrangement of a photosynthetic unit (LH1 and LH2 are localized around the reaction center (RC). (**a**) LH1 + RC Rhodospirillum rubrum growing at high light inten-sities, (**b**) LH1 + RC + LH2 Rhodopseudomonas palustris growing at low light intensities, (**c**) LH1 + RC + LH2 Rhodopseudomonas palustris growing at high light intensities, (**d**) LH1 + RC + LH2 Al-lochromatium minutissimum growing at low light intensities (data from Solovev and Erokhin, 2008 [86]).

The light-harvesting complexes of purple sulfur bacteria consist of two distinct complexes. The smaller peripheral LH2 complex (light-harvesting) transfers the absorbed energy to the larger internal LH1 complex, which passes the energy on to the reaction center (RC). The peripheral light-harvesting complex in the model organism *Allochromatium vinosum* consists of 12 copies of two short polypeptides called α and β, each of which has one α-helix passing through the membrane. The α and β polypeptides form two concentric protein cylinders in the membrane, between which are light-harvesting bacteriochlorophylls and carotenoids, 24 molecules of bacteriochlorophyll *a*, one molecule for each α and β is firmly attached to these polypeptide rings, twelve other bacteriochlorophyll molecules and is only weakly attached. The complex further contains light-harvesting carotenoids, such as spirilloxanthin. Internal complex of LH1 bacteria *Allochromatium vinosum* is composed of 2 α and 2 β polypeptide complexes arranged in a circle around the reaction center [86,87]. The model of a possible arrangement of a photosynthetic unit in species of *Rhodospirillum*, *Rhodopseudomonas* and *Allochromatium* genera is presented in Figure 5.

### 5.2. Reaction Centers of the Chromatiaceae

In purple sulfur bacteria, the reaction centers are located in specially modified parts of the inner layer of the plasma membrane called intracytoplasmic membranes. These areas are formed by invagination of the plasma membrane and take on a tubular or vesicular shape [88]. The reaction centers of purple sulfur bacteria consist of one copy of each, of three or four subunits, depending on the species studied. Subunits occurring in all species are referred to as light (L), medium (M) and severe (H). If a given species has four subunits, it is referred to as cytochrome ***c*** subunit. Interestingly, the names of the first three subunits do not correspond to their molar mass, as they were divided on the basis of mobility by SDS-PAGE [89]. In addition, the reaction centers contain other non-covalently bound cofactors, such as pigments, quinones or metal ions. In the model organism *Allochromatium vinosum* there are 4 molecules of bacteriochlorophyll *a*, two molecules of bacteriopheophytin, ubiquinone (also coenzyme Q_10_), menaquinone (Vitamin K_2_), metal ion Fe^2+^ and carotenoid, in this case, spirilloxanthin [90].

### 5.3. Structure of Chlorosomes

Chlorosomes are characteristic of representatives of green sulfur bacteria, in particular the *Chlorobiaceae* family. These structures are ovoid structures between 70–180 nm in length and 30–60 nm in width attached to the inner side of the plasma membrane. Inside there are about 10^5^ molecules of bacteriochlorophyll *c*, *d* or *e* aggregated together with a small amount of carotenoids and quinones. Around the chlorosomes there is probably a protein–lipid layer forming on the side connected to the plasma membrane the so-called baseplate complex, which binds bacteriochlorophyll *a* and other carotenoids. Chlorosomes are unique mainly in that their function is determined by pigment–pigment interactions, in contrast to pigment-protein interactions typical of, for example, purple bacteria [2,91].

**Figure 6:**
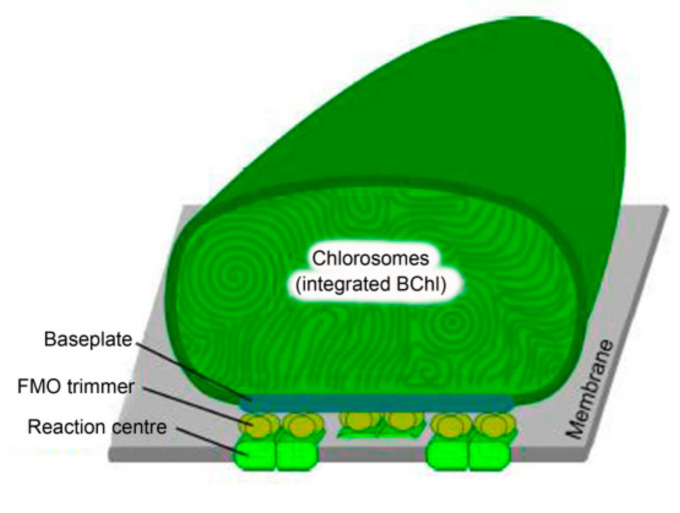
Schematic structure of the photosynthetic apparatus of green sulfur bacteria (the scheme modified from Dostál, 2014 [91].

The energy obtained by chlorosomes is transferred to the reaction center by means of a Fenna–Matthews–Olson protein. It is a water-soluble trimer that binds eight molecules of bacteriochlorophyll *a* and mediates the transfer of charge between the baseplate complex and the reaction center (Figure 6).

### 5.4. The Reaction Center of the Chlorobiaceae Family

The reaction center contains several membrane proteins, two copies of the pigment-binding protein PscA and one copy of PscB, 16 molecules of bacteriochlorophyll *a*, four chlorophylls and two carotenoids evenly distributed among the copies of PscA protein, which probably forms symmetric homodimers. Most of the bound bacteriochlorophyll *a* serve as a light collector [2,11].

The reaction center of the *Chlorobiaceae* is very similar to the reaction center of photosystem 1, which may be found in phototrophic oxygenations, but its exact molecular structure is not yet known and is largely derived from similarities. The reaction center contains several membrane proteins, two copies of pigment binding protein PscA and one copy of PscB, 16 molecules of bacteriochlorophyll *a*, four chlorophyll *a* and two carotenoids evenly distributed among the copies of PscA protein, most likely forming symmetrical homodimers. All excitation energy ends at a special pair of connected bacteriochlorophylls and, where it is converted to chemical energy, by a charge separation process [2,91,92].

## 6. Conclusions

Based on the general characteristics, representatives of the families *Chlorobiaceae* and *Chromatiaceae* were described, which differ in the number of representatives, the molecular and biochemical mechanism of anoxygenic photosynthesis and the photosynthetic apparatus. The universal electron donor for both families is reduced sulfur compounds, which are oxidized in the process of photosynthesis to elemental sulfur granules deposited inside (*Chromatiaceae*) or outside (*Chlorobiaceae*) cells. Another feature by which families are distinguished is the shape and size of the cells, the formation of cell aggregates, and also the color of the cell suspension, which is given by the pigment. The phylogeny and taxonomy of phototrophic microorganisms are based on their morphological, physiological and biochemical properties, as well as on the sequence analysis of the 16S rDNA gene and specific genes to their photosynthetic apparatus. For the *Chromatiaceae*, the taxonomic system corresponds to phylogenetic knowledge, while the system of the taxon *Chlorobiaceae* is still based on easily observable signs of classical taxonomy. *Allochromatium vinosum* is the most popular species for researchers of anoxygenic phototrophs and the most thoroughly studied at the genetic level.

Sulfur metabolism serves to oxidize reduced sulfur compounds to obtain electrons for anoxygenic photosynthesis and energy for bacterial growth. Various representatives may use various reduced sulfur compounds and oxidation mechanisms for this purpose. The mechanisms of sulfur oxidation are described for only a few representatives. Some of these mechanisms have not been experimentally demonstrated and are based only on similarities with other sulfur bacteria.

The photosynthetic apparatus of the families described above and their representatives differ; in particular, the photosynthetic pigments of the *Chromatiaceae* are located in the intracytoplasmic membranes. In contrast, in the taxon *Chlorobiaceae*, these pigments have specific structures called chlorosomes, which are located below the plasma membrane. Another difference between these families are pigments. *Chromatiaceae* possess Bchl *a* or *b* and the carotenoids, such as spirlloxanthine, rhodopine, lycopene, which stain cells from dark to light red. The *Chlorobiaceae* group contains Bchl *c*, *d* or *e* and the carotenoids chlorobactein, isorenieratin and β-carotene. The structure of chlorosome and light-harvesting complex 2 is relatively well described, while the structures of reaction centers and light-harvesting complex 1 are not. This is especially true for the family *Chromatiaceae*, which has not been studied in this respect at all, and most of the findings are based on similarities with other purple bacteria. Although we already know a lot about these families, further research is needed to fully understand all the processes, especially from structural biology and biochemistry. Another area that research could focus on is the application of already known mechanisms and products of these bacteria.

Today, these bacteria are only used in the treatment of wastewater and the production of biopolymers or H_2_, although other uses are already theoretically known. Examples are the production of carotenoids for industrial purposes or the possibility of using these bacteria to remove undesirable sulfur compounds from liquids or gases.

## Data Availability

Not applicable.
